# Distinct *Trypanosoma cruzi* isolates induce activation and apoptosis of human neutrophils

**DOI:** 10.1371/journal.pone.0188083

**Published:** 2017-11-27

**Authors:** Luísa M. D. Magalhães, Agostinho Viana, Augusto C. de Jesus, Egler Chiari, Lúcia Galvão, Juliana A. Gomes, Kenneth J. Gollob, Walderez O. Dutra

**Affiliations:** 1 Laboratório de Biologia das Interações Celulares, Departamento de Morfologia, Instituto de Ciências Biológicas, Universidade Federal de Minas Gerais, Belo Horizonte, Minas Gerais, Brazil; 2 Laboratório de Biologia do *Trypanosoma cruzi* e doença de Chagas, Departamento de Parasitologia, Instituto de Ciências Biológicas, Belo Horizonte, Minas Gerais, Brazil; 3 Núcleo de Ensino e Pesquisa, Instituto Mario Pena, Belo Horizonte, Minas Gerais, Brazil; 4 BRISA Diagnósticos, Belo Horizonte, Minas Gerais, Brazil; 5 AC Camargo Cancer Center, International Center for Research, São Paulo, São Paulo, Brazil; 6 INCT-DT, Belo Horizonte, Minas Gerais, Brazil; Albert Einstein College of Medicine, UNITED STATES

## Abstract

Neutrophils are critical players in the first line of defense against pathogens and in the activation of subsequent cellular responses. We aimed to determine the effects of the interaction of *Trypanosoma cruzi* with human neutrophils, using isolates of the two major *discrete type units* (DTUs) associated with Chagas’ disease in Latin America (clone Col1.7G2 and Y strain, DTU I and II, respectively). Thus, we used CFSE-stained trypomastigotes to measure neutrophil-*T*. *cruzi* interaction, neutrophil activation, cytokine expression and death, after infection with Col1.7G2 and Y strain. Our results show that the frequency of CFSE+ neutrophils, indicative of interaction, and CFSE intensity on a cell-per-cell basis were similar when comparing Col1.7G2 and Y strains. Interaction with *T*. *cruzi* increased neutrophil activation, as measured by CD282, CD284, TNF and IL-12 expression, although at different levels between the two strains. No change in IL-10 expression was observed after interaction of neutrophils with either strain. We observed that exposure to Y and Col1.7G2 caused marked neutrophil death. This was specific to neutrophils, since interaction of either strain with monocytes did not cause death. Our further analysis showed that neutrophil death was a result of apoptosis, which was associated with an upregulation of TNF-receptor, TNF and FasLigand, but not of Fas. Induction of TNF-associated neutrophil apoptosis by the different *T*. *cruzi* isolates may act as an effective common mechanism to decrease the host’s immune response and favor parasite survival.

## Introduction

Polymorphonuclear neutrophil granulocytes play an important role in the first line of defense against pathogens and the activation of subsequent immune responses [1]. The bone marrow of a healthy adult produces up to 10^11^ neutrophils per day, which can be increased during acute inflammation. These cells represent more than 50% of circulating leukocytes [2]. Neutrophils are the first cells recruited to infection sites and are important for host defense [1, 3–5]. These cells also provide an important link between innate and adaptive immunity during parasite infections [6,7].

Activated neutrophils have a short lifespan and undergo constitutive apoptosis. Removal of apoptotic neutrophils by macrophages turns off production of pro-inflammatory mediators and stimulates production of anti-inflammatory cytokines [8,9]. The importance of apoptosis in the modulation of immune responses in parasitic infections has been reported, showing that parasites such as *Toxoplasma gondii*, *Plasmodium falciparum*, and *Entamoeba histolytica*, are capable of inducing apoptosis in host cells to control the immune response [10–14].

Despite the importance of neutrophils in controlling infection by different parasites [15,16], little is known about the role of these cells in human *Trypanosoma cruzi* infection. It has been shown that human neutrophils can destroy intracellular forms of *T*. *cruzi* and that this activity is increased in the presence of colony-stimulating factor [17,18]. In addition, neutrophils from indeterminate Chagas disease patients display lower cytokine production after *in vitro* stimulation with *T*. *cruzi* antigens, compared with neutrophils from cardiac Chagas patients and non-infected individuals [19].

Biological and genetic variability within the *T*. *cruzi* population has led to the classification of the parasite population into six distinct *discrete type units* (DTUs) [20]. In addition to intrinsic differences, parasites belonging to different DTUs present distinct (although sometimes overlapping) geographic distribution, as well as association with different clinical forms [21]. Recent studies have demonstrated that trypomastigotes from different DTUs have distinct effects in immunological characteristics of human monocytes [22]. Isolates from TcI and TcII DTUs activate human monocytes, increasing expression of CD282 (TLR-2) and CD284 (TLR-4), as well as CD80 and cytokines [20]. Given that neutrophils are the most abundant immune cell found in human blood and critical players in the immune response, our goal was to evaluate the effects of the interaction with trypomastigotes belonging to the two main DTUs associated with Chagas’ disease in Latin America, TcI (Col1.7G2) and TcII (Y), in immunological characteristics of human neutrophils.

Our results showed that the percentage and intensity of interactions between human neutrophils and the different strains was similar, and that the interaction led to activation of neutrophils, as measured by expression of CD282, CD284 and IL-12. Moreover, interaction with both isolates led to a decreased viability of neutrophils but not monocytes. Interaction with Col1.7G2 and Y strain also induced a higher percentage of TNF, TNF-receptor and Fas Ligand expression by neutrophils, with no changes in Fas expression. These results show that Col1.7G2 and Y strain induce activation of human neutrophils, which may influence the subsequent immune response, but also induce apoptosis of these cells, possibly representing an escape mechanism common to the different *T*. *cruzi* strains, favoring parasite survival.

## Materials and methods

### Human samples

The donors included in our studies were non-Chagas healthy individuals (n = 9), as determined by negative specific serological tests for Chagas’ disease. Individuals were from Belo Horizonte city, state of Minas Gerais, Brazil, with average ages ranging between 23 and 34 years of age. They were recruited between January 2012 and January 2013. We excluded from our study individuals with any chronic inflammatory disease, diabetes, heart and circulatory illnesses (including hypertension) or bacterial infections. All individuals included in this work were volunteers and provided written informed consent. This work was approved by the Ethical Committee of the Universidade Federal de Minas Gerais, under the protocol# ETIC077/06. Peripheral blood was collected from the donors by venipuncture.

### Parasites

Tissue culture-derived trypomastigotes (TCT) of Col1.7G2 and Y strain were isolated from infected monolayers of LLC cells (from ATCC). LLC cells were infected using a ratio of five TCT: one host cell, and kept in DMEM enriched with 1% inactivated fetal calf serum (FCS), supplemented with antibiotics (penicillin at 500μ/mL and streptomycin at 0.5 mg/mL). After approximately 5 days, the TCT were collected from the supernatant, washed once by centrifugation with phosphate-buffered saline (PBS) pH 7.2 at 1000 x g for 10 min at 4°C, and resuspended in RPMI enriched with 5% of inactivated human serum, antibiotics (penicillin at 500U/mL and streptomycin at 05 mg/mL) and 1mM of L-glutamine to a concentration of 6x10^7^ TCT/mL. Parasites obtained in this manner were used for infecting peripheral blood cells from donors and adherent cells to continue the *in vitro* cycle. Contamination with amastigotes was always below 10%, as determined by light microscopy evaluation of all cultures.

### *T*. *cruzi* interaction with peripheral blood cells in suspension and flow cytometry

Trypomastigotes from LLC cultures, obtained as described above, were labeled with CFSE (carboxyfluorescein diacetate succinimidyl ester–Molecular Probes C1157), using a protocol previously reported by us [23], with modifications, and incubated with peripheral blood. Briefly, 6 x 10^7^ parasites were incubated with 5μM CFSE for 15 min at 37°C, 5% CO_2_. Labeled parasites were washed three times with cold PBS + 10% of inactivated FCS by centrifugation at 1000 x g for 10 min at 4°C. It has been previously demonstrated by our group that both strains stain similarly with CFSE [22].

Peripheral blood infections were performed with CFSE-labeled parasite in a proportion of 10 parasites/cell and incubated for 3 hours at 37°C in 5% CO_2_. After the incubation period, cells were washed by centrifugation with PBS at 600 x g for 10 min at 4°C to remove extracellular parasites and re-incubated for additional 12 hours with RPMI enriched with 5% of inactivated human serum, antibiotics (penicillin at 500U/mL and streptomycin at 05 mg/mL) and 1mM of L-glutamine. Brefeldin A (1μg/mL) was added for the last four hours of infection to prevent protein secretion. After incubation cells were washed with PBS by centrifugation at 600 x g for 10 min at 4°C. At the end of the centrifugation, the erythrocytes were lysed using RBC “Lysing buffer” (Bio Legend, CA, USA), at a concentration of 20mL/1mL of peripheral blood, by incubating for 15 min at 20°C in the dark. After the incubation, cells were washed three times by centrifugation and resuspended with PBS.

### Analysis of expression of surface molecules and cytokines by peripheral blood using flow cytometry

After lysis of erythrocytes, cells were immunostained and analyzed using multiparametric flow cytometry. We used a combination of monoclonal antibodies directed to surface molecules (PE-labeled CD282, CD284, and Annexin; PeCy7-labeled CD120b; APC-labeled CD14; BV421-Fas-L) and 7AAD. Intracellular cytokine expression and FAS expression were evaluated using PE labeled antibodies against Fas, TNF, IL-12/IL-23p40 and IL-10. For surface and intracellular straining, cells were treated as previously described by us [23]. After staining, cells were resuspended in PBS and acquired using a FACSCanto II (Becton & Dickinson, San Jose, CA, USA). An average of 100,000 events were acquired for each experiment. The analyses were performed using FlowJo 7.6.5 software (Tree Star Inc., Ashland, OR, USA).

**Fig 1:**
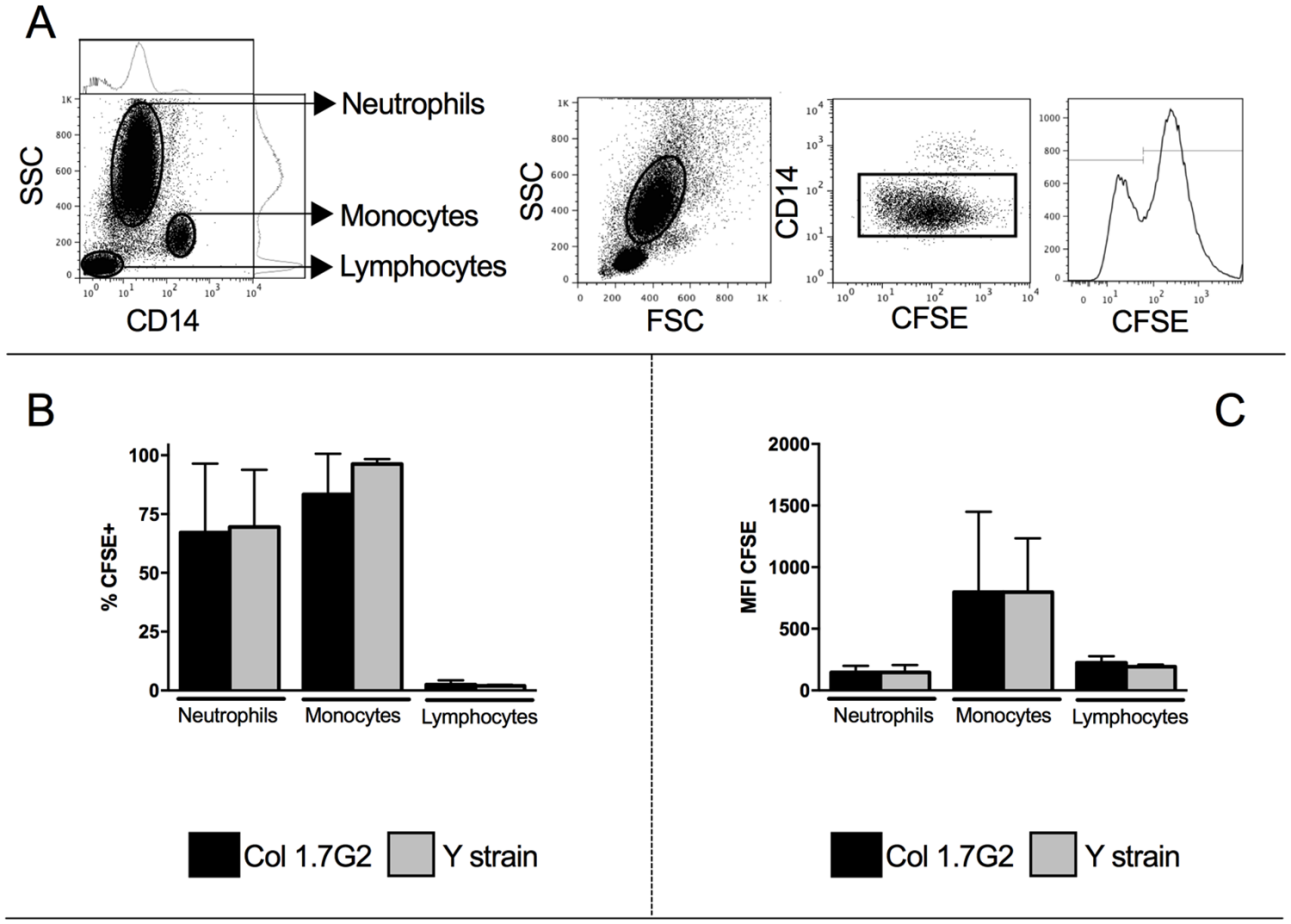
Evaluation of the frequency and intensity of CFSE+ neutrophils exposed to Y and Col1.7G2 trypomastigotes. Trypomastigotes from Col1.7G2 and Y strain were previously stained with CFSE and exposed *in vitro* to human neutrophils for 15 hours (n = 7) (A) The first panel shows a representative figure of CD14 expression *versus* granularity indicating that neutrophils express low CD14, and defining neutrophil, monocyte and lymphocyte gate. The second to fourth panels show representative plots of gating strategy for defining the neutrophil population. The second panel shows the selection of granulocytes in the size *versus* granularity (SSCxFSC), the third panel shows the selection of CD14low population, corresponding to neutrophils, and the fourth panel shows the histogram of CFSE and the selection of CFSE- and CFSE+ cells. (B) Percentage of CFSE+ neutrophils, monocytes and lymphocytes. (C) Mean intensity of expression of CFSE fluorescence by neutrophils, monocytes and lymphocytes. Results are expressed as averages and standard deviation. Comparisons between different strains were performed using Paired T test.

Previous studies have shown that neutrophils present a low expression of CD14 (CD14low), whereas monocytes express higher intensity of CD14 (CD14high) [24,25]. Thus, selection of the neutrophil population was made according to this strategy, by selecting granulocytes, based on FSC vs SSC plot, followed by selection of CD14low cells (Fig 1A). After selecting neutrophils, we gated on CFSE+ and CFSE- neutrophils, to evaluate the influence of parasite infection on the different parameters.

### Statistical analysis

All samples were submitted to ROUT test to identify outliers. We compared our results using One-Way Anova or Kruskal-Wallis, as indicated by the results obtained from running the Kolmogorov-Smirnov normality test. Correlation analyses were done using Pearson’s correlation coefficient. All analyses were performed using Graph Pad Prism Software (LaJolla, CA, USA). Differences that returned *p* values equal or less than 0.05 were considered statistically significant from one another.

## Results

### Col1.7G2 and Y strain trypomastigotes interact with human neutrophils, and do so with similar intensity

Trypomastigotes obtained from cell cultures were previously labeled with CFSE in order to determine the rate of interaction of the different *T*. *cruzi* isolates with human neutrophils. We observed a similar frequency of CFSE+ neutrophils after exposure to Col1.7G2 or Y strain in 15 hour cultures (Fig 1B). The intensity of infection on a cell per cell basis, as measured by the mean fluorescence intensity (MFI) of CFSE was also similar comparing neutrophils exposed to the different strains (Fig 1C). As previously shown by us (22), the frequencies and intensity of infection by human monocytes with either strain is similar (Fig 1B and 1C). The frequency of CFSE+ lymphocytes is much lower than neutrophils or monocytes (Fig 1B).

### Interaction with Col1.7G2 and Y strain increases CD282 and CD284 expression by human neutrophils

**Fig 2:**
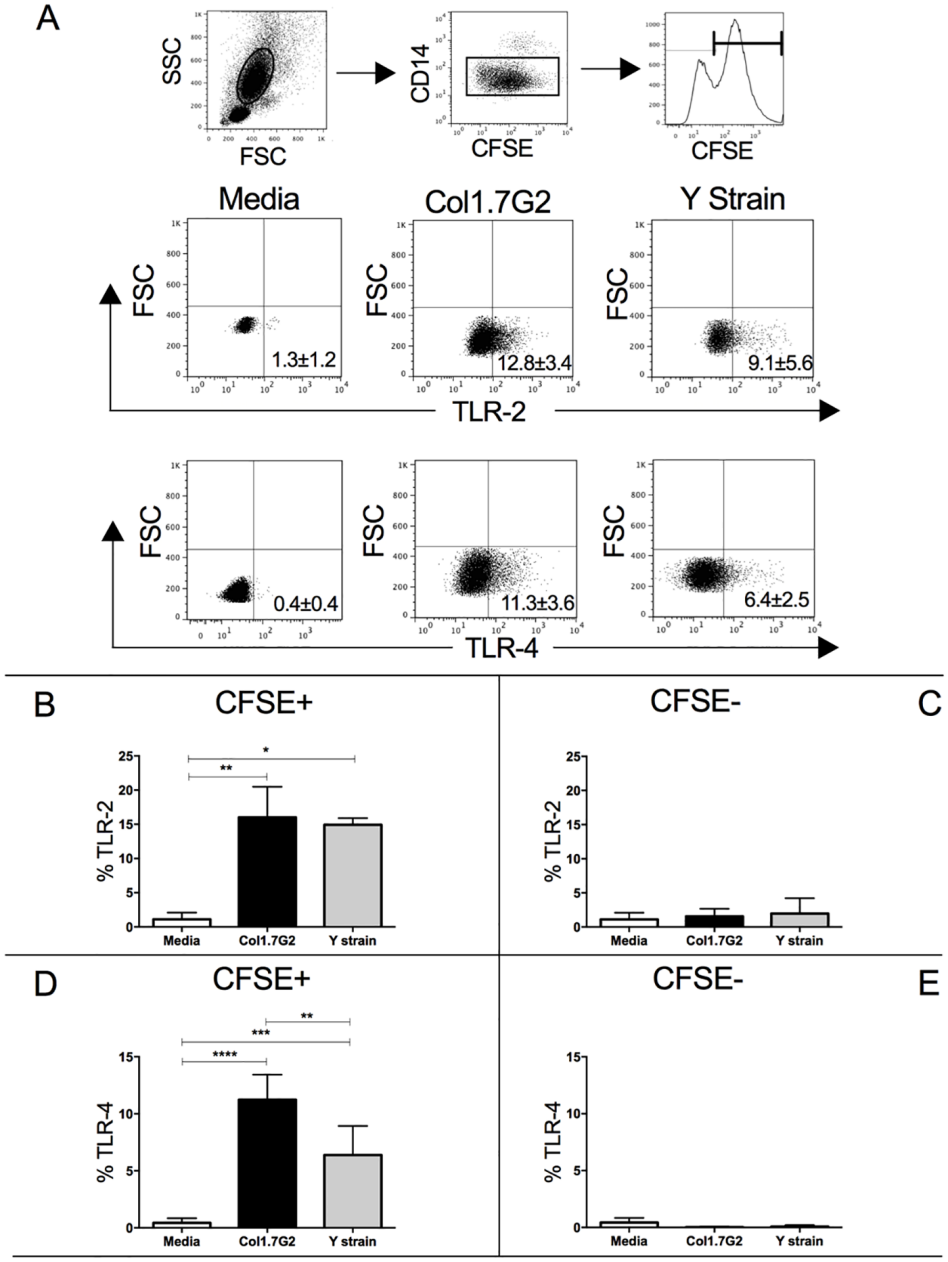
Expression of TLR-2 and TLR-4 by CFSE+ and CFSE- neutrophils exposed to different *T*. *cruzi* isolates. (A) Representative dot-plots of TLR-2 and TLR-4 expression by CFSE+ cells. First panel shows the selection of granulocytes in the size *versus* granularity (FSCxSSC), second panel shows the selection of CD14low population, corresponding to neutrophils. Third panel shows the histogram of CFSE, selecting the CFSE+ population, where the representative plots below were obtained. The lower panels show the selection of TLR-2 or TLR-4 positive cells *versus* forward scatter (FSC) from CFSE+ gated cells in media, as well as in cultures infected with different isolates. Similar strategy was used to define the expression of TLR-2 and TLR-4 in CFSE- neutrophils. (B) Percentage of TLR-2 in CFSE+ neutrophils; (C) Percentage of TLR-2 in CFSE- neutrophils; (D) Percentage of TLR-4 in CFSE+ neutrophils; (E) Percentage of TLR-4 in CFSE- neutrophils. Results are expressed as average ± standard deviation (n = 7). The symbols *, ** and **** indicate p < 0.05, p < 0.01 and p<0.0001 between groups, respectively. Comparisons between groups were performed using One-Way Anova (Panel D) or Kruskal-Wallis test (panels B, C and E) according to Kolmogorov-Smirnov normality test.

Previous studies have shown that neutrophil activation can be accompanied by increased expression of Toll-like receptors [24,26]. Thus, in order to investigate the activation of neutrophils after *in vitro* exposure to Col1.7G2 and Y strain, we assessed the expression of CD282 and CD284 by flow cytometry. Fig 2A shows the gating strategy for the analysis of CD282 and CD284 expression. Interaction with both strains led to a higher CD282 and CD284 expression (Fig 2B and 2D). A higher expression of CD284 was observed by interaction with Col1.7G2 compared to Y strain (Fig 2D). Increases in CD282 and CD284 were dependent on the parasite interaction, since CFSE- cells did not up regulate these molecules (Fig 2C and 2E).

### Interaction with Col1.7G2 and Y strain trypomastigotes increased IL-12 and TNF, but not IL-10, expression by human neutrophils

**Fig 3:**
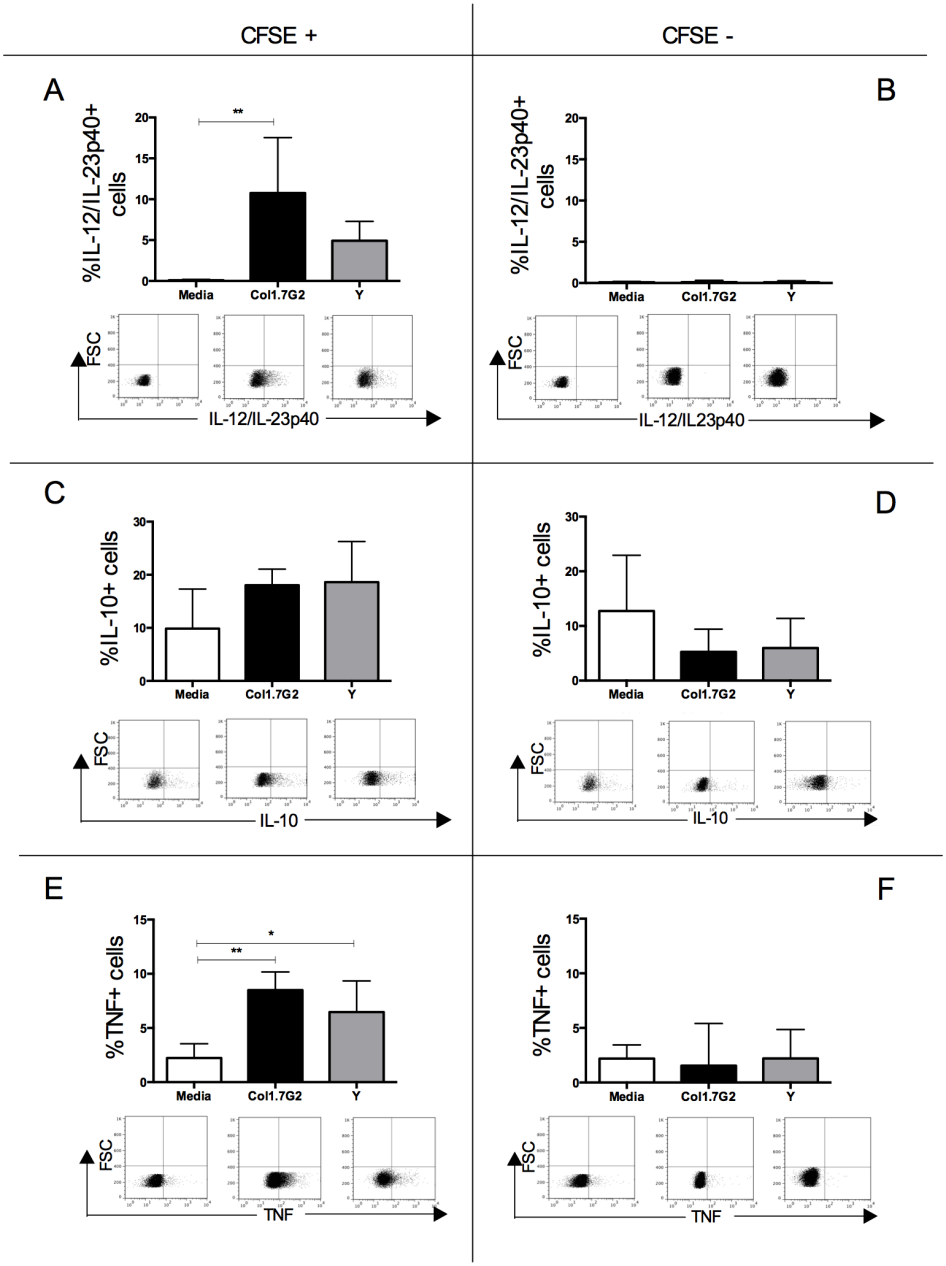
Cytokine expression by CFSE+ and CFSE- neutrophils exposed to different *T*. *cruzi* isolates. Determination of percentage of: (A) IL-12/IL-23p40 in CFSE+ neutrophils; (B) IL-12/IL-23p40 in CFSE- neutrophils; (C) IL-10 in CFSE+ neutrophils; (D) IL-10 in CFSE- neutrophils; (E) TNF in CFSE+ neutrophils; (F) TNF in CFSE- neutrophils. Results are expressed as average ± standard deviation (n = 7). The symbols * and ** indicate p < 0.05 and p < 0.01 between groups, respectively. The plots show the selection of IL-12/IL-23p40 or IL-10 or TNF positive cells *versus* forward scatter from CFSE+ and CFSE- gated cells in media, as well as cultures infected with different isolates. Comparisons between groups were performed using One-Way Anova or Kruskal-Wallis test according to Kolmogorov-Smirnov normality test.

We then questioned whether interaction with the different parasite strains and subsequent activation influenced the expression of cytokines by human neutrophils. It was observed that interaction with Col1.7G2, but not Y strain, led to a significant increase in expression of IL-12/IL-23p40 in CFSE+ neutrophils (Fig 3A). Infection with either Col1.7G2 or Y strain did not change the expression of IL-10 (Fig 3C). A higher expression of TNF in CFSE+ neutrophils in relation to non-infected culture was observed after contact with both isolates (Fig 3E). No change in the expression of cytokines was found in CFSE- neutrophils after exposure to either isolate (Fig 3B, 3D and 3F). Moreover, cytokine expression was lower in CFSE-, as compared to CFSE+ neutrophils (Fig 3, comparing left and right panels), and induction of IL-12 was totally dependent on parasite presence, since CFSE- neutrophils did no express any IL-12.

### Col1.7G2 and Y strain trypomastigotes decrease viability of human neutrophils and induce apoptosis in these cells

**Fig 4:**
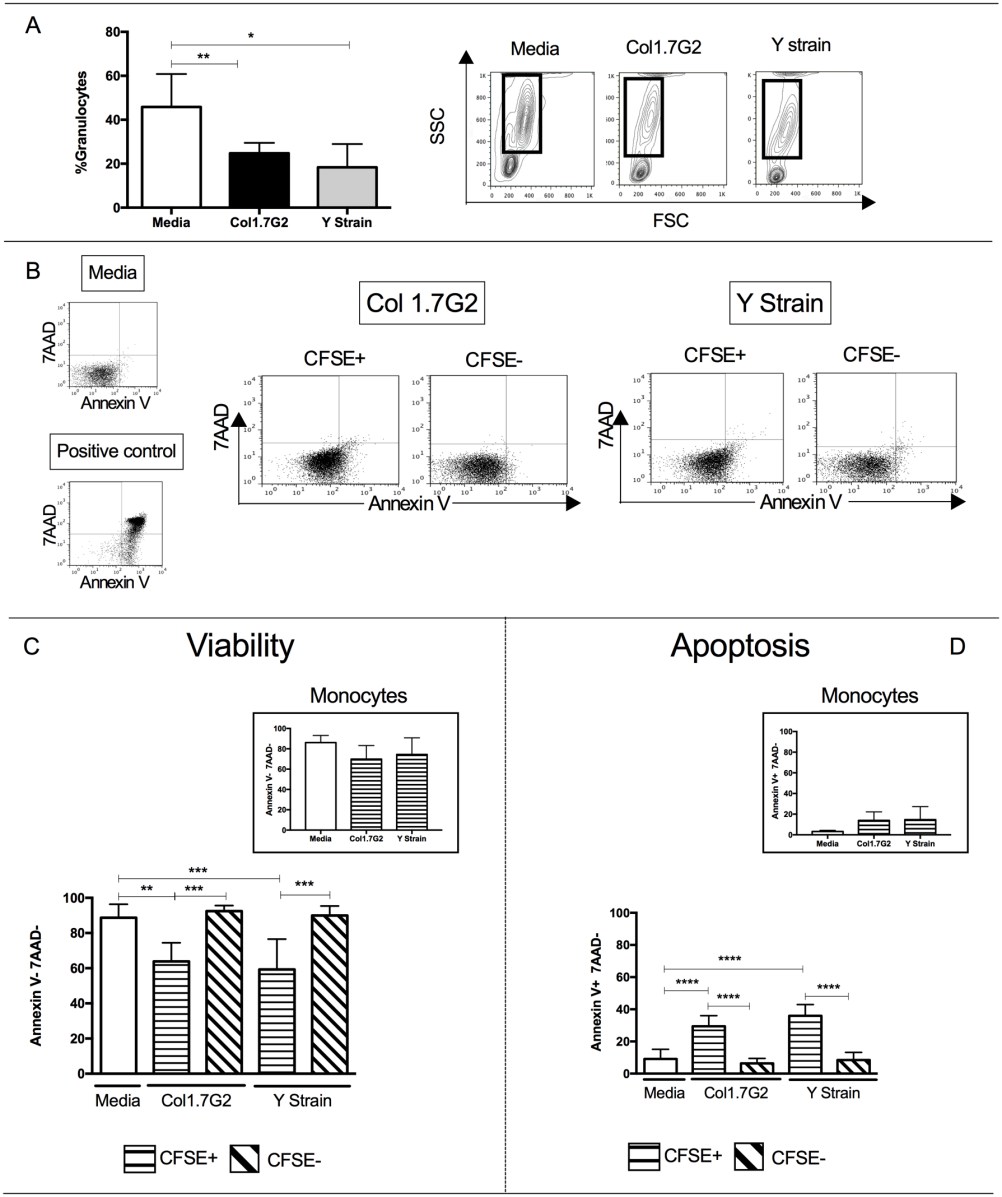
Evaluation of the viability of neutrophils exposed to trypomastigotes of Col1.7G2 and Y strain of *T*. *cruzi*. Trypomastigotes from Y strain and Col1.7G2 were previously stained with CFSE and incubated with peripheral blood for 15 hours, after which the frequency of granulocytes was accessed (n = 6). (A) Percentage of granulocytes and representative plots of size *versus* granularity (FSCxSSC), showing the gating of granulocytes. (B) Representative plots showing 7AAD *versus* AnnexinV in media, positive control, as well as cultures exposed to different isolates in CFSE+ and CFSE- neutrophils. (C) Frequency of neutrophils in media, and CFSE+ and CFSE- that do not express Annexin V or 7AAD (viable cells); insert shows viability of monocytes exposed to the different strains. (D) Frequency of neutrophils in media, and CFSE+ and CFSE- that express Annexin V but not 7AAD (apoptotic cells); insert shows apoptosis of monocytes exposed to the different strains. Results are expressed as average ± standard deviation. The symbols *, **, *** and **** indicates p < 0.05, p < 0.01, p < 0.001 and p<0.0001 between groups, respectively. Comparisons between groups were performed using One-Way Anova test according to Kolmogorov-Smirnov normality test.

The life span of circulating neutrophils is typically short (8-20h), but certain factors such as recruitment to an inflammation site and phagocytosis of infectious agents may change their lifespan [27,28]. In our study, we observed that the percentage of granulocytes decreased after 15 hours of exposure to both Col1.7G2 and Y strain when compared to media control (Fig 4A). We then evaluated the expression of Annexin V and 7AAD to determine cell viability and access the occurrence of apoptosis of neutrophils. Gating strategy for the analysis of 7AAD and Annexin V expression is shown in Fig 4B. We observed that the decrease in the granulocyte population was associated with a decreased viability of infected cells, since the frequency of viable cells (annexinV-7AAD-) was lower within the CFSE+ population (Fig 4C). This decrease in viability was not observed in monocytes exposed to either strain (Fig 4C, upper right panel).

To determine whether the observed viability reduction was associated with the occurrence of apoptosis, we determined the frequency of Annexin V+ 7AAD- cells and observed that the frequency of CFSE+ neutrophils, indicative of interaction with the different isolates, that underwent apoptosis (%AnnexinV+7AAD-) was significantly higher than CFSE- neutrophils (Fig 4D). Again, despite the fact that monocytes also get infected with trypomastigotes from either strain (Fig 1), they do not undergo apoptosis (Fig 4D, upper right panel).

### CFSE+ neutrophils exposed to Col1.7G2 and Y strain express higher percentage of TNF-receptor and FasL, but no significant changes in Fas expression

**Fig 5:**
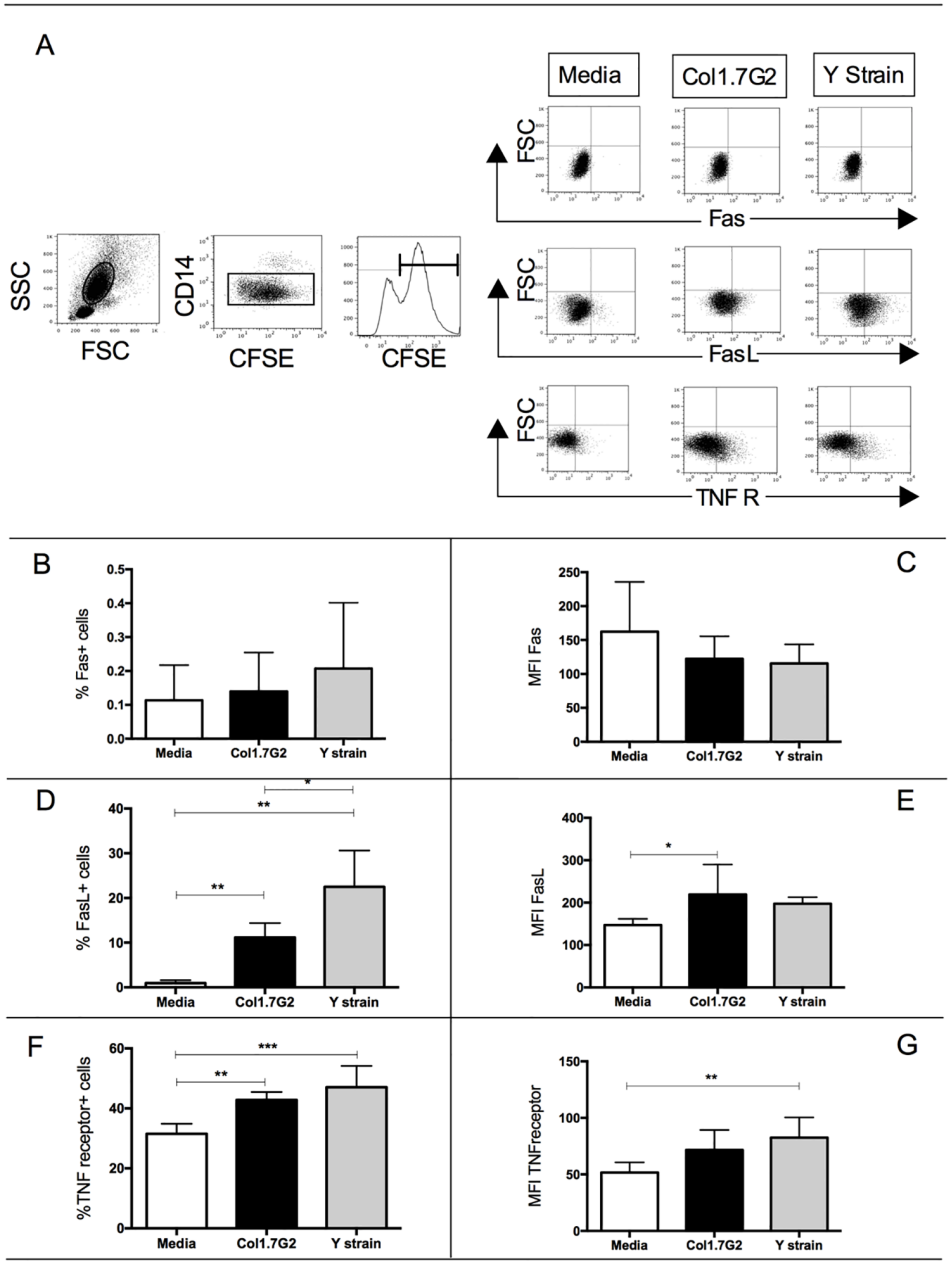
Expression of Fas, FasL and TNF-receptor in CFSE+ neutrophils exposed to different *T*. *cruzi* isolates. Trypomastigotes from Col1.7G2 and Y strain were previously stained with CFSE and exposed to peripheral blood *in vitro* for 15 hours, after which the percentage of expression of Fas, FasL and TNF-receptor in neutrophils were accessed (n = 6). (A) First panel shows the selection of granulocytes in the size *versus* granularity (FSCxSSC), second panel shows the selection of CD14low population, corresponding to neutrophils. Third panel shows the histogram of CFSE. The other panels show representative plots of the selection of Fas, FasL or TNF-receptor *versus* granularity in CFSE+ neutrophils. (B) percentage expression of Fas in CFSE+ neutrophils; (C) mean intensity of Fas expression in CFSE+ neutrophils; (D), percentage of expression of FasL in CFSE+ neutrophils; (E) mean intensity of FasL expression in CFSE+ neutrophils; (F) percentage of expression of TNF-receptor in CFSE+ neutrophils; (G) and mean intensity of expression of TNF-receptor in CFSE+ neutrophils. Results are expressed as average ± standard deviation. The symbols *, ** and *** indicate respectively *p* < 0.05, p<0.01 and p<0.001 between groups. Comparisons between groups were performed using One-Way Anova.

In order to investigate the pathway associated with apoptosis after exposure to Col1.7G2 and Y strain, we evaluated the expression of Fas and TNF-receptor II. Both molecules are related to the activation of caspase 8 and induction of apoptosis through the extrinsic pathway [29]. TNF R II has greater affinity for TNF and also a greater half-life of binding TNF than TNF R I [30]. Fig 5A shows the gating strategy used in the analysis of Fas, FasL and TNF receptor in CFSE+ neutrophils. Although interaction with Col1.7G2 and Y strain does not lead to a higher percentage or intensity of expression of Fas in CFSE+ neutrophils (Fig 5B and 5C), expression of FasL was upregulated in CFSE+ neutrophils (Fig 5D and 5E). Strikingly, interaction with both strains induced a higher percentage of expression of TNF-receptor in these cells (Fig 5F) and only Y strain induced higher intensity of expression of TNF-receptor (Fig 5G).

### Higher intensity of CFSE expression is correlated with higher TNF, IL-10 and apoptosis in neutrophils exposed to Y strain trypomastigotes

**Fig 6:**
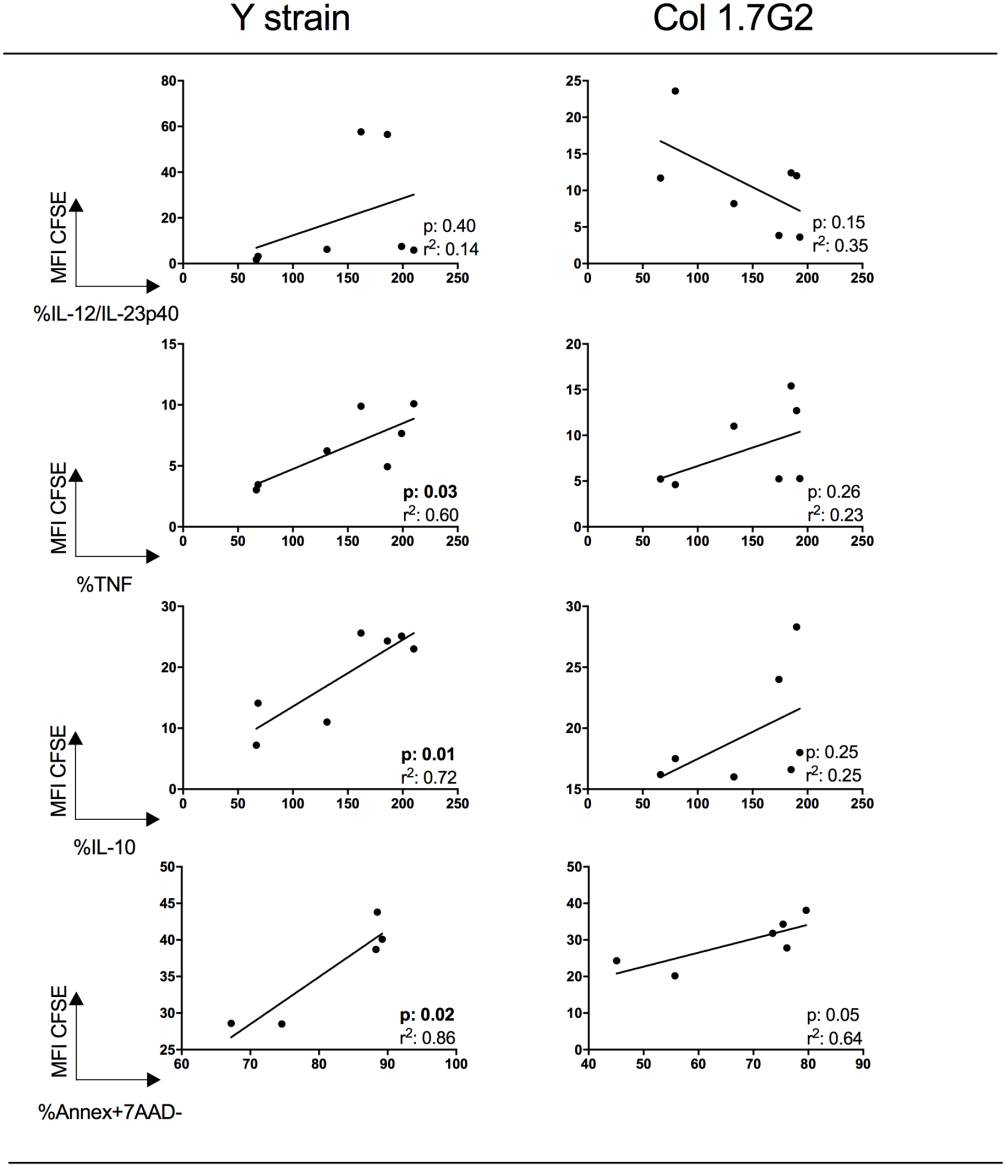
Correlative analysis between intensity of expression of CFSE and IL-12, TNF, IL-10 and apoptosis in neutrophils exposed to trypomastigotes from Y strain and Col1.7G2. Cells were incubated in the presence of CFSE-labeled trypomastigote forms of the different strains of *T*. *cruzi* and analyzed for CFSE intensity (MIF CFSE), IL-12+, TNF+, IL-10+ and annaexinV+7AAD-, as described in Material and Methods. Correlative analyses were performed with MIF CFSE (Y axis), and IL-12, TNF, IL-10 or annexinV+7AAD- (X axes). r^2^ as well as *p* values are indicated in each graph.

In order to determine if the intensity of neutrophil-trypomastigote interaction was related to cytokine expression and apoptosis, we performed correlation analysis between the frequency of neutrophils expressing IL-12, TNF or IL-10, as well as the frequency of annexinV+7AAD- cells, and the intensity of CFSE expression. We observed a statistically significant positive correlation between the frequencies of TNF, IL-10 and annexinV+7AAD- cells with mean intensity of CFSE expression after exposure to Y strain trypomastigotes, suggesting that the higher the parasite interaction, the higher the expression of these cytokines and the occurrence of apoptosis (Fig 6). No correlation was observed between IL-12 expression and CFSE intensity, and no statistically significant correlations were observed between the different parameters in cultures exposed to Col1.7G2 (Fig 6).

## Discussion

In this work, we demonstrated that *T*. *cruzi* trypomastigotes belonging to DTUs I and II (Col1.7G2 and Y strain, respectively) are capable of interacting with and activating human neutrophils, and induce their apoptosis. These DTUs are the main parasite groups associated with Chagas disease in Latin America and this mechanism may be important for parasite survival in the human host, by decreasing the immune response.

Our data showed that the frequency of CFSE+ neutrophils, indicative of interaction between neutrophil and trypomastigotes, as well as the intensity of CFSE expression on a cell-per-cell basis, were similar when comparing Y strain and Col1.7G2 (Fig 1B and 1C). Although monocytes are preferentially infected with *T*. *cruzi* [22], neutrophils have also been shown to internalize trypomastigotes [19,23]. Comparing the infection of neutrophils *versus* monocytes, we observed that the percentage of neutrophils infected by either isolate of *T*. *cruzi* was similar to the frequency of monocytes infected by the same strains (Fig 1B). However, the intensity of infection in neutrophils was significantly lower when compared to the values observed in human monocytes (Fig 1C). Sanderson and colleagues suggested that amastigotes of *T*. *cruzi* are capable of replicating in macrophages but not in neutrophils, as confirmed by the higher intensity of infection found in macrophages in that study [31]. This could explain the lower intensity of infection in neutrophils observed in this work, despite the short-term culture. Another possibility is that, although neutrophils and monocytes present a similar phagocytic capacity [32], it is known that the expression of CD282, is higher in the latter [24]. This difference in CD282 expression could be responsible for the lower intensity of neutrophil infection, since its activation is related to the internalization of *T*. *cruzi* [33]. Further studies are required to directly address this issue.

Our next goal was to evaluate the activation status of neutrophils after exposure to the different isolates. For this, we assessed the expression of CD282 and CD284 after exposure to trypomastigotes of Y strain and Col1.7G2. It has been shown that *T*. *cruzi* expresses several CD282 and CD284 agonists [34]. Both receptors are important in the activation of the innate immune system, and CD282 acts as an immunoregulator in the early stage of infection. CD282 (-/-) mice showed increased production of IL-12/IL-23p40 and IFN-gamma after *T*. *cruzi* infection [35]. CD282 activation has also been implicated in phagocytosis of trypomastigotes [36]. Moreover, CD284 expression is critical in the control of parasites during the acute phase. It has been shown that CD284 signaling triggers an important early parasiticidal event against *T*. *cruzi*, which is dependent on the formation of NO and ROS [37]. We observed an increased expression of CD282 and CD284 following exposure to either isolates, compared to media control. Bystander neutrophils (CFSE-) had a lower expression of CD282 and CD284 compared to CFSE+, demonstrating that expression of CD282 and CD284 was dependent on the presence of the parasite. Furthermore, exposure to Co1.7G2 led to a higher CD284 expression compared to infection with Y strain.

Since the increase in CD282 and CD284 expression in CFSE+ neutrophils indicates an activation of these cells, we sought to evaluate the expression of the immunoregulatory cytokines IL-12/IL-23p40, IL-10 and TNF. In agreement with the results of CD282 and CD284 expression, only CFSE+ neutrophils had an increased expression of cytokines. Interestingly, our data showed a statistically significant increase in IL-12/IL-23p40 expression only after infection with Col1.7G2, which could be related to higher CD284 expression, as previously suggested [34]. While TNF was increased in neutrophils exposed to both isolates, no alterations in IL-10 expression were observed. It was previously shown that infection with Col1.7G2 induces high expression of IL-10 by human monocytes, as compared to Y strain, and that this increase was not dependent on parasite contact, since it was observed in CFSE+ and CFSE- cells [19]. This shows that different cell types react differently to infection, possibly due to activation of distinct surface receptors and/or intracellular pathways.

Previous studies have shown that TNF is an apoptosis inducer in many different cell types [38,39]. It is noteworthy that the population of neutrophils in which we observed the higher rate of apoptosis (CFSE+) is also the one expressing higher TNF. TNF expression also triggers the formation of neutrophils extracellular traps (NET) [40], an important control mechanism of the innate immune response, elicited by different protozoa [16,41].

Our results showed a decrease in the percentage of granulocytes after interaction with the two *T*. *cruzi* isolates. We also demonstrated that CFSE+ neutrophils exposed to both Col1.7G2 and Y strain display a decrease in viability compared with CFSE- neutrophils (Fig 4). Similar results were found in murine neutrophils infected with *Leishmania amazonensis* [42]. On the other hand, infection of human peripheral blood neutrophils *in vitro* with *Leishmania major* has been shown to inhibit apoptosis [43]. Thus, induction of neutrophil apoptosis may play a role in some parasitic infections but not others. Decreased viability was not observed in monocytes, despite the fact that these cells are highly infected by these strains ([22] and Fig 1), suggesting that this is a specific activity of *T*. *cruzi* over neutrophils.

We found that neutrophils exposed to both isolates display higher percentage of apoptosis compared with non-infected ones. Interestingly, Freire-de-Lima and colleagues demonstrated that macrophages in contact with apoptotic cells favor the intracellular parasite growth and increase parasitemia [44,45]. More recently, it was demonstrated that apoptotic neutrophils increase *T*. *cruzi* replication in murine macrophages [11]. Therefore, the induction of neutrophil apoptosis by *T*. *cruzi* may enhance phagocytosis by monocytes and macrophages, favoring infection. It is also known that internalization and/or binding of apoptotic cells down-regulates the response of macrophages [9,44,46,47] and monocytes [48,49]. The induction of phagocytosis of apoptotic neutrophils also acts as an escape mechanism of the immune system since macrophages that have ingested apoptotic cells *in vitro* inhibit production of pro-inflammatory mediators [9,47,49].

In order to investigate whether Col1.7G2 and Y strain induce apoptosis in a similar manner, we evaluated the expression of Fas and TNF-receptor molecules. The Fas-FasL and TNFR-TNF binding induce apoptosis through activation of caspase 8 and 10, corresponding to the extrinsic pathway of apoptosis [29]. Lula and colleagues described a strong correlation between soluble TNF superfamily ligands and heart failure parameters in chronic Chagas patients presenting functional ventricular disturbances [50]. Strikingly, interaction with both isolates induced higher expression of TNF-receptor by neutrophils. Increased expression of TNF and its receptor is an indicative that both Col1.7G2 and Y strain induce apoptosis through this pathway, even though further studies are necessary to investigate whether *T*. *cruzi* infection induces apoptosis through other activation pathways as well. Another possibility would be the Fas-FasL pathway. While no differences were observed in Fas expression by neutrophils after infection, Fas-L was upregulated. However, given the very low expression of Fas by these cells (~0.2%), it is unlikely that this pathway plays an important function, favoring the TNF-mediated mechanism. The upregulation of FasL by both isolates indicates that these infected cells may induce apoptosis of other cell populations, also providing a mechanism of parasite survival. Cabral-Piccin and colleagues demonstrated that apoptotic CD8 lymphocytes favor M2 phenotype and *T*. *cruzi* replication in macrophages. They also showed that blocking apoptosis through anti-FasL treatment can restrict parasite growth and increase NO production [45]. Our analysis also showed that the higher the intensity of CFSE expression, indicating interaction with a higher number of parasites, the higher the expression of cytokines and the occurrence of apoptosis. Interestingly, this correlation was only statistically significant when performed with Y strain trypomastigotes and not with Col1.7G2 isolate. Previous studies have shown that acute Chagas patients and individuals with disease reactivation due to HIV infection or transplantation display leukopenia [51–53]. This suggests that neutropenia could indeed be occurring *in vivo*, presenting an important correlation with our data.

Taken together our results show that Tc I and Tc II *T*. *cruzi* isolates are capable of interacting and activating human neutrophils. Interaction with both isolates induces apoptosis, likely through the TNF-TNFreceptor extrinsic pathway. The induction of apoptosis may act as an effective escape mechanism of the host immune response. The fact that both isolates lead to a similar reduction of cell viability, rate of apoptosis, and expression of TNF and TNF-receptor in human neutrophils indicate that this may be a highly conserved mechanism for the species, since the isolates are representatives of the two most abundant DTUs, TcI (clone Col cl1.7) and TcII (Y strain). Although different DTUs exhibit different biological properties, it is known that there is some overlap between strains from different DTU. Moreover, heterogeneity is also observed amongst strains belonging to the same DTU. This finding inspires new studies of infection control by targeting parasite-neutrophil interaction.

Although this study provides important new information regarding neutrophil-*T*. *cruzi* interaction and its possible implications in the immune response and parasite survival, some limitations need to be taken into account. The exact apoptosis signaling and pathway still remain to be determined. Moreover, in order to truly validate the induction of apoptosis as a mechanism common to the species, it is critical to evaluate other isolates of the parasite, belonging to the same DTUs, as well as to other DTUs. This limitations open perspectives for further studies.

## References

[1] Yamashiro S, Kamohara H, Wang JM, Yang D, Gong WH, Yoshimura T. Phenotypic and functional change of cytokine-activated neutrophils: inflammatory neutrophils are heterogeneous and enhance adaptive immune responses. J Leukoc Biol. 2001;69(5):698–704. 11358976

[2] Laskay T, van Zandbergen G, Solbach W. Neutrophil granulocytes as host cells and transport vehicles for intracellular pathogens: Apoptosis as infection-promoting factor. Immunobiology. 2008;213(3–4):183–91. doi: 10.1016/j.imbio.2007.11.010 18406366 10.1016/j.imbio.2007.11.010

[3] Laskay T, van Zandbergen G, Solbach W. Neutrophil granulocytes-Trojan horses for *Leishmania major* and other intracellular microbes? Trends Microbiol. 2003 5;11(5):210–4. 12781523 10.1016/s0966-842x(03)00075-1

[4] Nauseef WM. How human neutrophils kill and degrade microbes: an integrated view. Immunol Rev. 2007 10;219:88–102. doi: 10.1111/j.1600-065X.2007.00550.x 17850484 10.1111/j.1600-065X.2007.00550.x

[5] Faurschou M, Borregaard N. Neutrophil granules and secretory vesicles in inflammation. Microbes Infect. 2003 11;5(14):1317–27. 14613775 10.1016/j.micinf.2003.09.008

[6] Appelberg R. Neutrophils and intracellular pathogens: beyond phagocytosis and killing. Trends Microbiol. 2007 2;15(2):87–92. doi: 10.1016/j.tim.2006.11.009 17157505 10.1016/j.tim.2006.11.009

[7] Nathan C. Neutrophils and immunity: challenges and opportunities. Nat Rev Immunol. 2006 3;6(3):173–82. doi: 10.1038/nri1785 16498448 10.1038/nri1785

[8] Huynh M-LN, Fadok VA, Henson PM. Phosphatidylserine-dependent ingestion of apoptotic cells promotes TGF-beta1 secretion and the resolution of inflammation. J Clin Invest. 2002 1;109(1):41–50. doi: 10.1172/JCI11638 11781349 10.1172/JCI11638 PMC150814

[9] Fadok VA, Bratton DL, Konowal A, Freed PW, Westcott JY, Henson PM. Macrophages that have ingested apoptotic cells in vitro inhibit proinflammatory cytokine production through autocrine/paracrine mechanisms involving TGF-beta, PGE2, and PAF. J Clin Invest. 1998 2;101(4):890–8. doi: 10.1172/JCI1112 9466984 10.1172/JCI1112 PMC508637

[10] Rodrigues V, Agrelli GS, Leon SC, Silva Teixeira DN, Tostes S, Rocha-Rodrigues DB. Fas/Fas-L expression, apoptosis and low proliferative response are associated with heart failure in patients with chronic Chagas’ disease. Microbes Infect. 2008;10(1):29–37. doi: 10.1016/j.micinf.2007.09.015 18078776 10.1016/j.micinf.2007.09.015

[11] Luna-Gomes T, Filardy A a., Rocha JDB, Decote-Ricardo D, LaRocque-de-Freitas IF, Morrot A, et al Neutrophils increase or reduce parasite burden in *Trypanosoma cruzi*-infected macrophages, depending on host strain: Role of neutrophil elastase. PLoS One. 2014;9(3):3–10. 10.1371/journal.pone.0090582 PMC3944110 24599360

[12] Bannai H, Nishikawa Y, Ibrahim HM, Yamada K, Kawase O, Watanabe J, et al Overproduction of the pro-apoptotic molecule, programmed cell death 5, in *Toxoplasma gondii* leads to increased apoptosis of host macrophages. J Vet Med Sci. 2009;71(9):1183–9. 19801898 10.1292/jvms.71.1183

[13] Kemp K, Akanmori BD, Hviid L. West African donors have high percentages of activated cytokine producing T cells that are prone to apoptosis. Clin Exp Immunol. 2001;126(1):69–75. doi: 10.1046/j.1365-2249.2001.01657.x 11678901 10.1046/j.1365-2249.2001.01657.x PMC1906163

[14] Becker SM, Cho K-N, Guo X, Fendig K, Oosman MN, Whitehead R, et al Epithelial cell apoptosis facilitates *Entamoeba histolytica* infection in the gut. Am J Pathol. American Society for Investigative Pathology; 2010;176(3):1316–22. 10.2353/ajpath.2010.090740 PMC2832152 20093500

[15] Ribeiro-Gomes FL, Otero AC, Gomes N A, Moniz-De-Souza MC, Cysne-Finkelstein L, Arnholdt AC, et al Macrophage interactions with neutrophils regulate *Leishmania major* infection. J Immunol. 2004;172(7):4454–62. 15034061 10.4049/jimmunol.172.7.4454

[16] Abi Abdallah DS, Denkers EY. Neutrophils cast extracellular traps in response to protozoan parasites. Front Immunol. 2012;3(DEC):1–6. 23248631 10.3389/fimmu.2012.00382 PMC3522045

[17] Villalta F, Kierszenbaum F. Role of polymorphonuclear cells in Chagas’ disease. I. Uptake and mechanisms of destruction of intracellular (amastigote) forms of *Trypanosoma cruzi* by human neutrophils. J Immunol. United States; 1983 9;131(3):1504–10. 6309964

[18] Villalta F, Kierszenbaum F. Effects of human colony-stimulating factor on the uptake and destruction of a pathogenic parasite (*Trypanosoma cruzi*) by human neutrophils. J Immunol. United States; 1986 9;137(5):1703–7. 3528288

[19] Gomes J A S, Campi-Azevedo A. C, Teixeira-Carvalho A., Silveira-Lemos D, Vitelli-Avelar D, Sathler-Avelar R, et al Impaired phagocytic capacity driven by downregulation of major phagocytosis-related cell surface molecules elicits an overall modulatory cytokine profile in neutrophils and monocytes from the indeterminate clinical form of Chagas disease. Immunobiology. Immunobiology; 2012;217(10):1005–16. doi: 10.1016/j.imbio.2012.01.014 22387073 10.1016/j.imbio.2012.01.014

[20] Tibayrenc M. Epidemiology of parasitic protozoa and other pathogens. Genet Epidemiol. 1999; 10.1146/annurev.genet.33.1.449 10690415

[21] Zingales B, Miles M A., Campbell D A., Tibayrenc M, Macedo AM, Teixeira MMG, et al The revised *Trypanosoma cruzi* subspecific nomenclature: Rationale, epidemiological relevance and research applications. Infect Genet Evol. Elsevier B.V.; 2012;12(2):240–53. 10.1016/j.meegid.2011.12.009 22226704

[22] Magalhães LMD, Viana A, Chiari E, Galvão LMC, Gollob KJ, Dutra WO. Differential activation of human monocytes and lymphocytes by distinct strains of *Trypanosoma cruzi*. PLoS Negl Trop Dis. 2015;9(7):e0003816 doi: 10.1371/journal.pntd.0003816 26147698 10.1371/journal.pntd.0003816 PMC4492932

[23] Souza PE a, Rocha MOC, Rocha-Vieira E, Menezes C a S, Chaves ACL, Gollob KJ, et al Monocytes from patients with indeterminate and cardiac forms of Chagas’ disease display distinct phenotypic and functional characteristics associated with morbidity. Infect Immun. 2004;72(9):5283–91. doi: 10.1128/IAI.72.9.5283-5291.2004 15322024 10.1128/IAI.72.9.5283-5291.2004 PMC517423

[24] Kurt-Jones E a, Mandell L, Whitney C, Padgett A, Gosselin K, Newburger PE, et al Role of toll-like receptor 2 (TLR2) in neutrophil activation: GM-CSF enhances TLR2 expression and TLR2-mediated interleukin 8 responses in neutrophils. Blood. 2002;100(5):1860–8. 12176910

[25] Antal-Szalmas P, Strijp J a, Weersink a J, Verhoef J, Van Kessel KP. Quantitation of surface CD14 on human monocytes and neutrophils. J Leukoc Biol. 1997;61(6):721–8. 9201263 10.1002/jlb.61.6.721

[26] Riise RE, Bernson E, Aurelius J, Martner A., Pesce S, Della Chiesa M, et al TLR-stimulated neutrophils instruct NK Cells to trigger dendritic cell maturation and promote adaptive T cell responses. J Immunol. 2015; 10.4049/jimmunol.1500709 26085684

[27] Luo HR, Loison F. Constitutive neutrophil apoptosis: mechanisms and regulation. Am J Hematol. 2008 4;83(4):288–95. doi: 10.1002/ajh.21078 17924549 10.1002/ajh.21078

[28] Colotta F, Re F, Polentarutti N, Sozzani S, Mantovani A. Modulation of granulocyte survival and programmed cell death by cytokines and bacterial products. Blood. 1992 10;80(8):2012–20. 1382715

[29] Blankenberg FG. In vivo detection of apoptosis. J Nucl Med. 2008;49 Suppl 2:81S–95S. 18523067 10.2967/jnumed.107.045898

[30] Tartaglia LA, Pennica D, Goeddels DV. Ligand Passing: The 75-kDa tumor necrosis factor (TNF) receptor recruits TNF for signaling by the 55-kDa TNF receptor. J Biol Chem. 1993;18542–8. 8395508

[31] Sanderson CJ, de Souza W. A morphological study of the interaction between *Trypanosoma cruzi* and rat eosinophils, neutrophils and macrophages in vitro. J Cell Sci. 1979;37:275–86. 383733 10.1242/jcs.37.1.275

[32] Silva MT, Correia-Neves M, Nyakeriga A, Tech T. Neutrophils and macrophages : the main partners of phagocyte cell systems. 2012;3(July):2008–13. 10.3389/fimmu.2012.00174 PMC3389340 22783254

[33] Aoki MP, Carrera-Silva EA, Cuervo H, Fresno M, Giroǹs N, Gea S. Nonimmune cells contribute to crosstalk between immune cells and inflammatory mediators in the innate response to *Trypanosoma cruzi* infection. J Parasitol Res. 2012;2012. 10.1155/2012/737324 PMC3159004 21869919

[34] Rodrigues MM, Oliveira AC, Bellio M. The immune response to *Trypanosoma cruzi*: Role of toll-like receptors and perspectives for vaccine development. J Parasitol Res. 2012;2012. 10.1155/2012/507874 PMC3306967 22496959

[35] Gravina HD, Antonelli L, Gazzinelli RT, Ropert C. Differential use of TLR2 and TLR9 in the regulation of immune responses during the infection with *Trypanosoma cruzi*. PLoS One. 2013;8(5). 10.1371/journal.pone.0063100 PMC3641106 23650544

[36] Bafica A, Santiago HC, Goldszmid R, Ropert C, Gazzinelli RT, Sher A. Cutting edge: TLR9 and TLR2 signaling together account for MyD88-dependent control of parasitemia in *Trypanosoma cruzi* infection. J Immunol. 2006;177(6):3515–9. 16951309 10.4049/jimmunol.177.6.3515

[37] Oliveira A-C, de Alencar BC, Tzelepis F, Klezewsky W, da Silva RN, Neves FS, et al Impaired innate immunity in TLR4(-/-) mice but preserved CD8+ T cell responses against *Trypanosoma cruzi* in TLR4-, TLR2-, TLR9- or Myd88-deficient mice. PLoS Pathog. 2010;6(4):e1000870 doi: 10.1371/journal.ppat.1000870 20442858 10.1371/journal.ppat.1000870 PMC2861687

[38] Brenner D, Blaser H, Mak TW. Regulation of tumour necrosis factor signalling: live or let die. Nat Rev Immunol. 2015 5;15(6):362–74. doi: 10.1038/nri3834 26008591 10.1038/nri3834

[39] Chaves AT, de Assis Silva Gomes Estanislau J, Fiuza JA, Carvalho AT, Ferreira KS, Fares RCG, et al Immunoregulatory mechanisms in Chagas disease: modulation of apoptosis in T-cell mediated immune responses. BMC Infect Dis. BMC Infectious Diseases; 2016;16(1):191. 27138039 10.1186/s12879-016-1523-1 PMC4852404

[40] Manda A, Pruchniak MP, Araźna M, Demkow UA. Neutrophil extracellular traps in physiology and pathology. Cent J Immunol / Polish Soc Immunol Elev other Cent Immunol Soc. 2014 1;39(1):116–21. 10.5114/ceji.2014.42136 PMC4439979 26155111

[41] Kruger P, Saffarzadeh M, Weber ANR, Rieber N, Radsak M, von Bernuth H, et al Neutrophils: between host defence, immune modulation, and tissue injury. PLOS Pathog. 2015;11(3):e1004651 doi: 10.1371/journal.ppat.1004651 25764063 10.1371/journal.ppat.1004651 PMC4357453

[42] Carlsen ED, Hay C, Henard C a., Popov V, Garg NJ, Soong L. Leishmania amazonensis amastigotes trigger neutrophil activation but resist neutrophil microbicidal mechanisms. Infect Immun. 2013;81(11):3966–74. doi: 10.1128/IAI.00770-13 23918780 10.1128/IAI.00770-13 PMC3811833

[43] Baron EJ, Proctor RA. Elicitation of peritoneal polymorphonuclear neutrophils from mice. J Immunol Methods. 1982 3;49(3):305–13. 7040554 10.1016/0022-1759(82)90130-2

[44] Freire-de-Lima CG, Nascimento DO, Soares MB, Bozza PT, Castro-Faria-Neto HC, de Mello FG, et al Uptake of apoptotic cells drives the growth of a pathogenic trypanosome in macrophages. Nature (Internet). 2000;403(6766):199–203. 10.1038/35003208 10646605

[45] Cabral-Piccin MP, Guillermo LVC, Vellozo NS, Filardy A, Pereira-Marques ST, Rigoni TS, et al Apoptotic CD8 T-lymphocytes disable macrophage-mediated immunity to *Trypanosoma cruzi* infection. Cell Death Dis. 2016;7(5):e2232. 27195678 10.1038/cddis.2016.135 PMC4917666

[46] Stern M, Savill J, Haslett C. Human monocyte-derived macrophage phagocytosis of senescent eosinophils undergoing apoptosis. Mediation by alpha v beta 3/CD36/thrombospondin recognition mechanism and lack of phlogistic response. Am J Pathol. 1996 9;149(3):911–21. 8780395 PMC1865155

[47] Freire-de-Lima CG, Xiao YQ, Gardai SJ, Bratton DL, Schiemann WP, Henson PM. Apoptotic cells, through Transforming Growth Factor-beta, coordinately induce anti-inflammatory and suppress pro-inflammatory eicosanoid and NO synthesis in murine macrophages. J Biol Chem. 2006 12;281(50):38376–84. doi: 10.1074/jbc.M605146200 17056601 10.1074/jbc.M605146200

[48] Byrne A, Reen DJ. Lipopolysaccharide induces rapid production of IL-10 by monocytes in the presence of apoptotic neutrophils. J Immunol. 2002 2;168(4):1968–77. 11823533 10.4049/jimmunol.168.4.1968

[49] Voll RE, Herrmann M, Roth EA, Stach C, Kalden JR, Girkontaite I. Immunosuppressive effects of apoptotic cells. Nature. 1997 11;390(6658):350–1. doi: 10.1038/37022 9389474 10.1038/37022

[50] Lula JF, Otavio M, Pinho L, Pereira C, Teixeira MM, Bahia MT. Plasma concentrations of tumour necrosis factor-alpha, tumour necrosis factor-related apoptosis-inducing ligand, and FasLigand / CD95L in patients with Chagas cardiomyopathy correlate with left ventricular dysfunction. Eur J Heart Fail. 2009;825–31. doi: 10.1093/eurjhf/hfp105 19654138 10.1093/eurjhf/hfp105

[51] Pinto AYN, Ferreira AG Jr, Valente VC, Harada GS, Valente SAS. Urban outbreak of acute Chagas disease in Amazon region of Brazil: four-year follow-up after treatment with benznidazole. Rev. Pan Salud Publica 2009;25:77–83. 10.1590/s1020-49892009000100012 19341528

[52] Ferreira MS, Nishioda SA, Silvestre MTA, Borges AS, Nunes-Araújo FRF, Rocha A. Reactivation of Chagas’ Disease in Patients with AIDS: Report of Three New Cases and Review of the Literature. Clin Infect Dis 1997;25:1397–1400. 9431385 10.1086/516130

[53] Riarte A, Luna C, Sabatiello R, Sinagra A, Schiavelli R, De Rissio A, et al Chagas disease in patients with kidney transplant: 7 years of experience, 1989–1996. Cin Infect Dis 1999;29:561–567. 10.1086/598634 10530448

